# Supplementary material for: Visualizing nanometric structures with sub-millimeter waves
Source: Nat Commun. 2021 Dec 7;12:7091. doi: 10.1038/s41467-021-27264-x (PMC8651651; doi:10.1038/s41467-021-27264-x)
Supplement: Supplementary file 1 — Supplementary Information [file 41467_2021_27264_MOESM1_ESM.pdf]

Visualizing nanometric structures with sub-millimeter waves – Supplementary material

Alonso Ingar Romero, Amlan kusum Mukherjee, Anuar Fernandez Olvera, Mario Mendez Aller, and Sascha Preu

Department of Electrical Engineering and Information Technology, Technical University Darmstadt,

64283 Darmstadt, Germany

Corresponding authors. Email: amlan.mukherjee@tu-darmstadt.de, preu@imp.tu-darmstadt.de

Supplementary material

SPEED

The speed of the demonstrated system is limited by (I) the acquisition time of the spectra, (II) the use of only a single photoconductive pixel and (III) the data evaluation. The single spectrum acquisition time of 24 s (6 ms integration time for 4000 frequency points) can be increased by 1-2 orders of magnitude by replacing the DFB laser diode system with a fast sweep system[1], where 24 spectra per second with 8750 data points have been recorded. A major bottle neck is the use of a single photoconductive receiver, requiring a 2D scan of the object. Instead, the receiver can be replaced by a second p-i-n-diode-based Terahertz emitter with several tens of microwatt local oscillator power in order to drive a state-of-the-art Terahertz camera, such as a microbolometer[2] or a field effect transistor-based camera[3]. Mixing of the local oscillator signal with the signal transmitted through the object will yield a homodyne mixing term similar to that of a classic Mach Zehnder interferometer or in holographic imaging techniques[4]. The mixing term contains all information required to perform the Hilbert-transform-based algorithm. In this way, the amount of pixels is solely limited by the pixel number of the camera, without the requirement of a mechanical scan. Last but not least, the data evaluation routines can be programmed onto a field programmable gate array (FPGA) which may finally enable real-time image processing.

CORRELATION OF MEASUREMENT PRECISION WITH OPTICAL PATH LENGTH DIFFERENCE

Fig. S1 shows the measured rms height error for various total optical path length differences $\Delta(nd)$ or, alternatively, homodyne fringe periods $\Delta f_{THz} = \frac{c_0}{\Delta(nd)}$ as shown in Fig. 1b. Since $\Delta(nd)$ is approximately known from geometry (though with a considerable error), we can estimate the fluctuations of the laser system on the time scale of the frequency sweep (24 s) from Eq. (13) using the slopes shown in Fig. S1. They are estimated to be around δf_{THz} ≈209±50 kHz, a value supported by the spectral analysis of the emitted Terahertz signal. This is in the range of the laser linewidth but considerably smaller than the smallest frequency step size used in the measurements, i.e. 40 MHz.

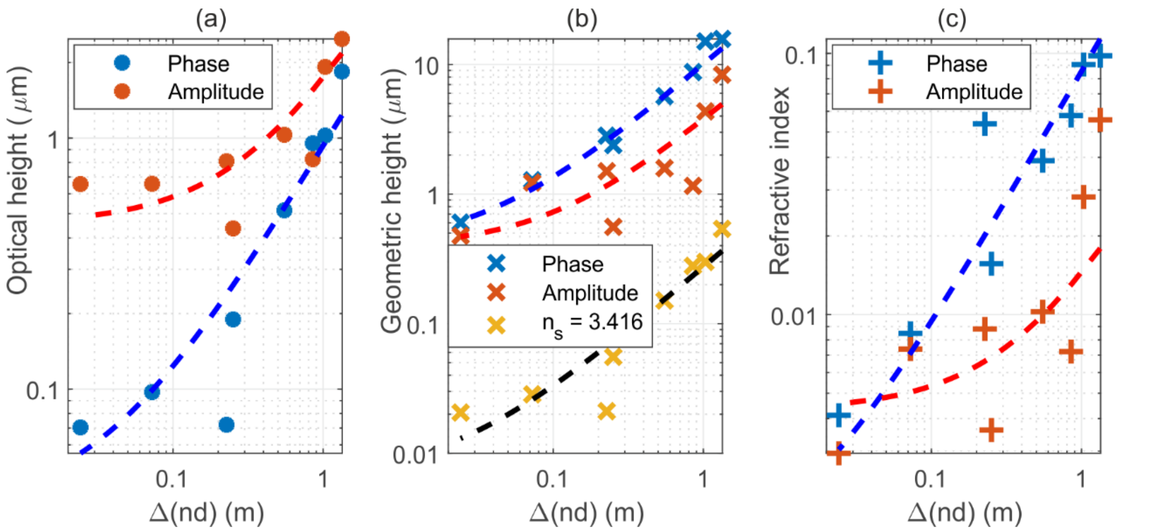

Fig. S1: The rms error for: a) optical thickness, b) geometric height and c) refractive index for different optical path length differences $\Delta(nd)$. The error of parameter estimation is calculated using either the amplitude or the phase information of the Fabry-Pérot oscillations. The dashed line shows the fitted values for the rms errors, assuming saturation determined by Eq. (14) at low noise levels and a linear increase at large $\Delta(nd)$ determined by Eq. (13). Blue/red fits are for the cases in which the refractive index and the thickness are evaluated simultaneously, whereas the yellow/black are for the cases when height is calculated directly from the optical thickness using the Fabry-Pérot oscillations and assuming a constant refractive index of the sample.

LIMTIATIONS OF THE FABRY-PÉROT CAVITY-ENHANCED IMAGING

For fitting the Fabry-Pérot resonances, the rms noise ϑ_{rms} given by Eq. (11) must be smaller than the visibility of the Fabry-Pérot oscillations of the phase V_ϑ. V_ϑ is given by the difference between the maximum and minimum phase change caused by the Fabry-Pérot resonances, hence requiring

$$V_\vartheta = 2\arctan\frac{R\sqrt{1-R^2}}{1-R^2} > \frac{2\pi\Delta(nd)}{c_0}\delta f_{THz} = \vartheta_{rms} \qquad \text{(S1)}$$

For the concrete example of a silicon wafer with reflectivity $R = 0.30$, $V_\vartheta = 0.609$ rad. For the case of Teflon with $R = 0.034$, $V_\vartheta = 0.0675$ rad. In practice, however, perturbations caused by standing waves in the setup (with phase amplitudes around 0.26 rad) exceed ϑ_{rms} by orders of magnitude, showing that it is difficult to apply the Fabry-Pérot method to Teflon while it works well for silicon.

OTHER NOISE SOURCES

Phase noise by incoherence: The homodyne Terahertz imager uses a coherent detection technique. If the total path length difference of the interferometer arms $\Delta(nd)$ approaches the coherence length, the visibility of the homodyne fringes (see Fig. 1b)) will be reduced and further phase fluctuations will arise. In the given experiment, the path length difference was below 0.6 m for all measurements. This is much shorter than the self-heterodyne (i.e. short-term) coherence length and still 1-2 orders of magnitude shorter than the long term coherence length.

Amplitude noise caused by the limited dynamic range and the lasers' intensity fluctuations. Its contribution, however, is fairly marginal for the DNRs between 30 and 40 dB used in the experiment.

Phase noise by undesired reflections in the setup: Undesired reflections in the setup, such as the ones arising between the uncoated TPX lenses, the sample and the various optical components in the setup, as well as within the silicon lens of the source or receiver, will also cause Fabry-Pérot-like oscillations that are superimposed on the Fabry-Pérot oscillations caused by the sample. Typically, their oscillation period is much shorter than that caused by the sample, as the distance of the reflecting objects is much

larger. These oscillations may partly be reduced with filtering prior to fitting. Still, they severely impact the height resolution.

SAMPLES CONSISTING OF TWO LAYER STRUCTURES

In the manuscript, we only considered the total optical thickness of the structure on the silicon wafer, irrespective of whether the Siemens star was made of the same material as the substrate (Fig. 2) or made of different materials (SiN and SiC on silicon as in Fig. 3). In the following, we evaluate the error caused by this simplification. Fig. S2 shows the physically correct structure.

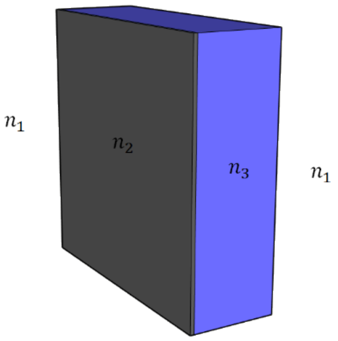

Fig. S2: Geometry of the two layer resonator.

Using the formalism presented in ref. [5], the field transmission coefficient of a two-layer loss-less FP resonator is found to be

$$t_{tot} = \frac{t_{12}t_{23}t_{31}e^{jk_0(n_2d_2+n_3d_3)}}{1+r_{12}r_{23}e^{j2k_0n_2d_2}+r_{12}r_{31}e^{j2k_0(n_2d_2+n_3d_3)}+r_{23}r_{31}e^{j2k_0n_3d_3}}$$

Where r_{ij} and t_{ij} are the reflection and transmission coefficients when going form interface i to interface j, and n_2, n_3, d_2 and d_3 are the refractive indices and the thicknesses of material 2 and material 3, respectively. $k_0 = 2\pi f_{THz}/c_0$ is the vacuum wavenumber.

For $r_{23} \approx 0$ and $t_{23} \approx 1$, the equation reduces to the single-layer FP resonator. This is the case for the Si-SiC interface, where the difference between the phase calculated with the single-layer model and the exact value is less than 0.0005 rad, resulting in an underestimation of the thickness by 1.83 nm (using $n_2 = 3.416$, $n_3 = 3.1$ [6], $d_2 = 525$ μm, $d_3 = 49$ nm and a frequency range of 600-800 GHz). We remark that the refractive index of SiC is afflicted by large errors as CVD-deposited material may deviate from its crystalline counterpart or the reported measurements in the literature[6]. The actual comparison between the used approximation and the exact phase is shown in Fig. S3, the error is plotted in Fig. S4.

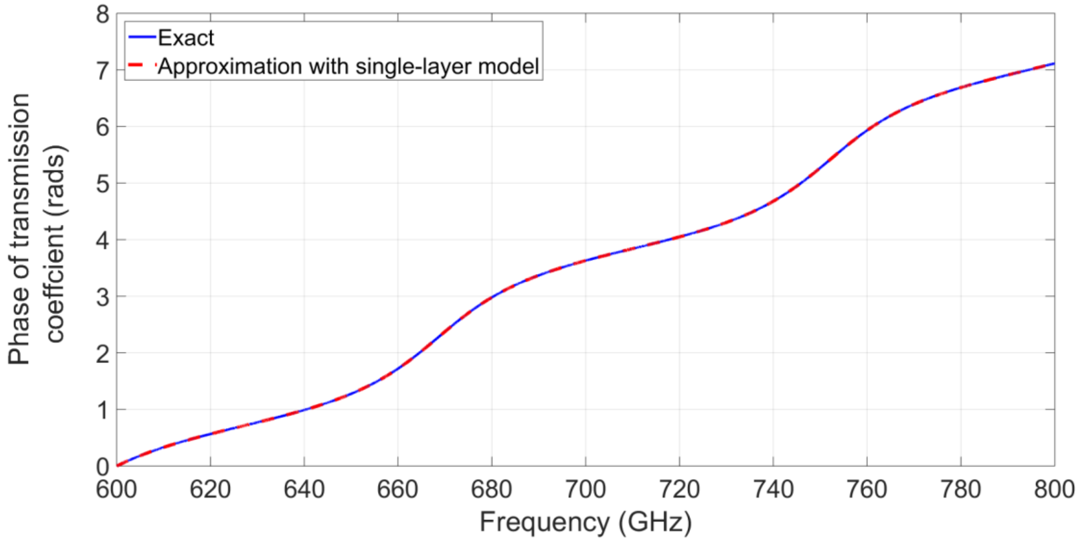

Figure S3. Comparison of the phase of the transmission coefficient as given by exact solution and by the single-layer approximation for the Si-SiC interface (n_2 = 3.416, n_3 = 3.1, d_2 = 525 μm, d_3 = 49 nm).

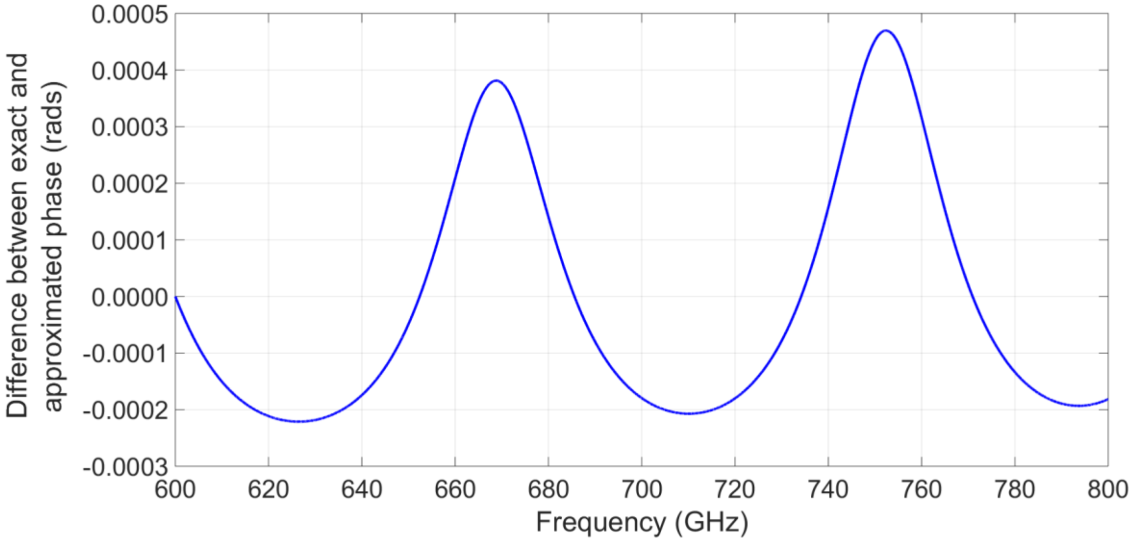

Figure S4. Difference between the transmitted phase estimated by the single-layer approximation and the exact value for the Si-SiC resonator (n_2 = 3.416, n_3 = 3.1 [6], d_2 = 525 μm, d_3 = 49 nm).

A similar result is found for the Si-SiN interface, but in this case the difference between the phases increases to a maximum of around 0.002 rad, resulting in an overestimation of the thickness equal to 1.98 nm (using $n_2 = 3.416$, $n_3 = 2.75$ [7], $d_2 = 508$ μm, $d_3 = 250$ nm and a frequency range of 600-800 GHz). The errors are summarized in Fig. S5 and S6. The latter also includes the absolute thickness error of SiC on silicon due to the single layer approximation.

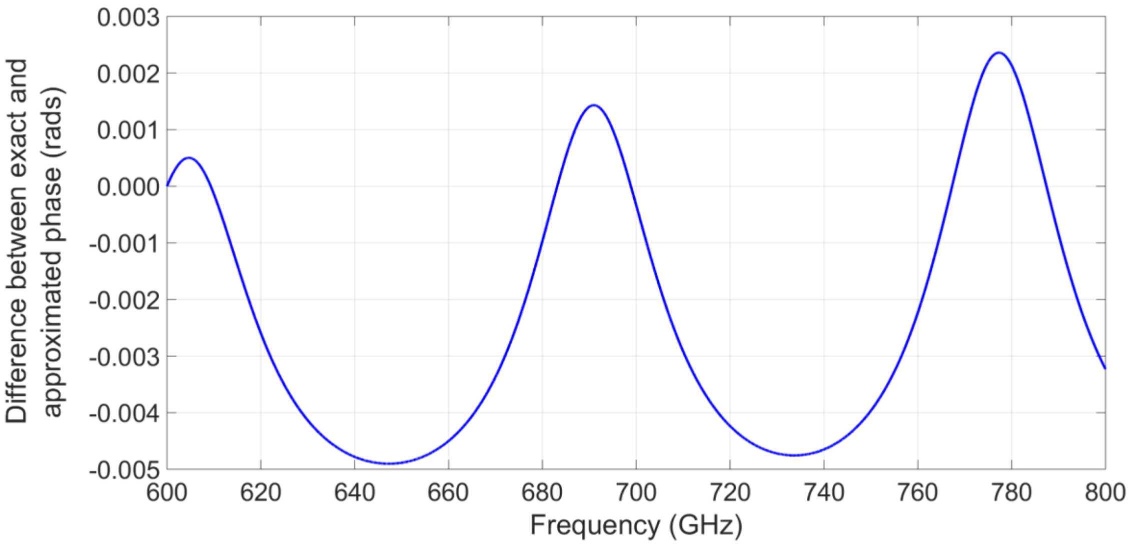

Figure S5. Difference between the transmitted phase estimated by the single-layer approximation and the exact value for the Si-SiN resonator (using $n_2 = 3.416$, $n_3 = 2.75$, $d_2 = 508$ μm, $d_3 = 250$ nm).

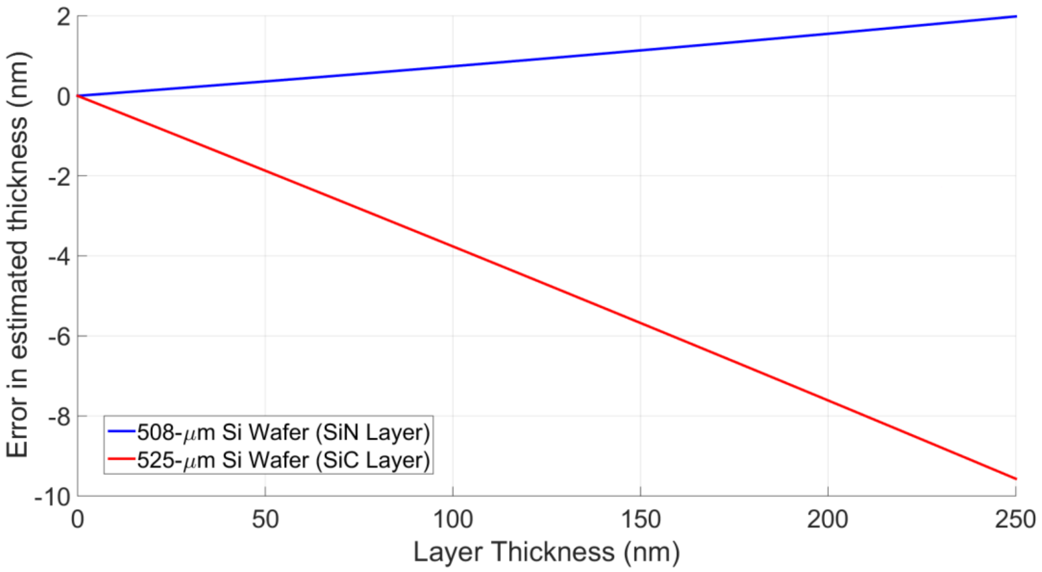

Figure S6. Absolute error (defined as estimated thickness minus actual thickness) in the estimated thickness as a function of the layer thickness for the case of a SiN layer grown on a 508 μm silicon wafer, and a SiC layer grown in a 525 μm silicon wafer.

FURTHER CHARACTERIZATION EXAMPLES

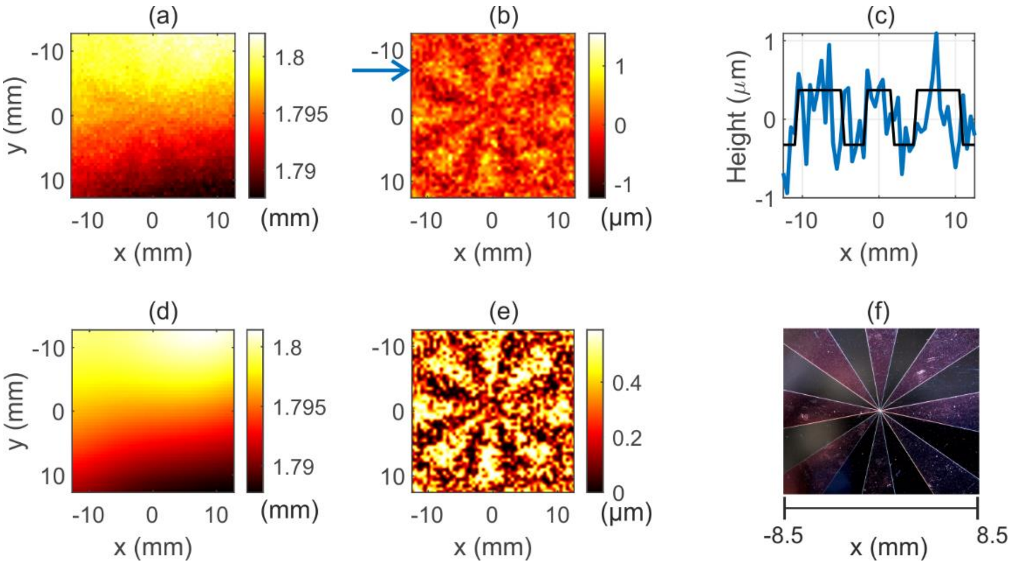

Fig. S7: a) Raw image data (51x51 pixels) of a 350 nm thick Siemens star of SiC on a 525 ± 5 μm thick silicon wafer (optical thickness of 1.795 mm) measured with a large optical path length difference of $\Delta(nd) = 55.16$ cm. Warping of the substrate is clearly visible with an amplitude of about 4 μm. 350 nm thick Siemens star not yet visible. b) Height profile after subtraction of warped background. c) Cross section through the line indicated by the blue arrow in b). The black step profile with an optical height of 695 nm serves as a guide for the eye. The rms error of the optical height profile is 402 nm. d) Warped background. e) Image enhanced by clipping and scaling height profile data in b). Siemens star is now clearly visible. f) Optical micrograph of the 350 nm SiN Siemens star.

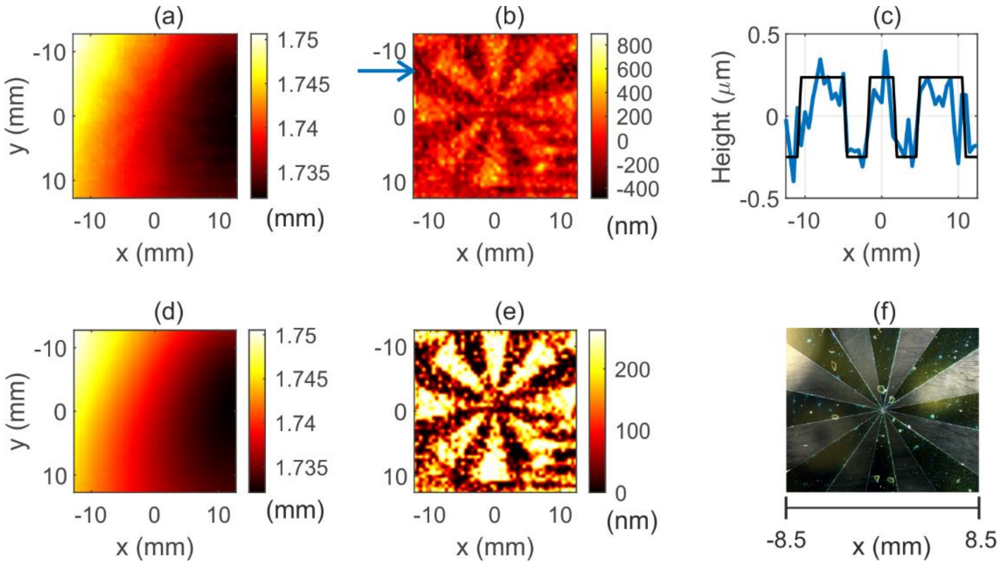

Fig. S8: a) Raw image data (51x51 pixels) of a 250 nm thick Siemens star of SiN on a 509 ± 5 μm thick silicon wafer (optical thickness of 1.739 mm) measured with an optical path length difference $\Delta(nd) = 7.25$ cm. Warping of the substrate is clearly visible with an amplitude of about 5 μm. 250 nm thick Siemens star is not yet visible. b) Height profile after subtraction of warped background. c) Cross section through the line indicated by the blue arrow in b). The black step profile serves as a guide for the eye. The rms error of the optical thickness is 160 nm, corresponding to a physical thickness variation of about 78 nm. d) Warped background. e) Contrast-enhanced image and scaled height profile data in b). Siemens star is now clearly visible. f) Optical micrograph of the 250 nm SiN Siemens star.

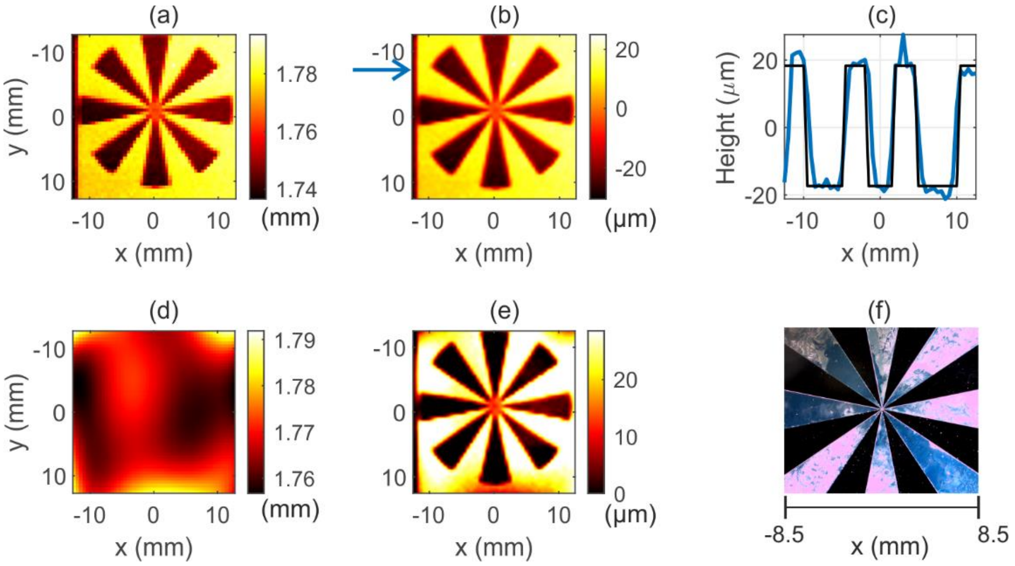

Fig. S9: a) Raw image data (51x51 pixels) of a 10.7 µm Siemens star etched into a silicon wafer of 520 $\pm$ 5 µm thickness (optical thickness of 1.776 mm) measured with an optical path length difference $\Delta(nd)$ = 55.16 cm. Here, the Siemens star's height is already larger than the amplitude of the warping and therefore already visible in the raw data. b) Interpolated and Gaussian filtered raw data with 100 times increased number of pixels. c) Cross-section through the line indicated by the blue arrow in b). The black step profile with an optical height of 35.9 µm serves as a guide for the eye. The optical thickness matches very well the expected value of 36.55 µm. d) Over-estimated warped background. The over-estimation of the warping originates from the fact that the structure is larger than the warping and part of the correction algorithm. Positions with little structure (edges and corners, e.g.) are therefore over-corrected. e) Image of the Siemens star after removing the warped background from raw data and image enhancement. The image enhancement steps are unnecessary for high Δh as it tends to overcorrect the image. f) Optical micrograph of the 10.7 µm etched Siemens star under white light illumination.

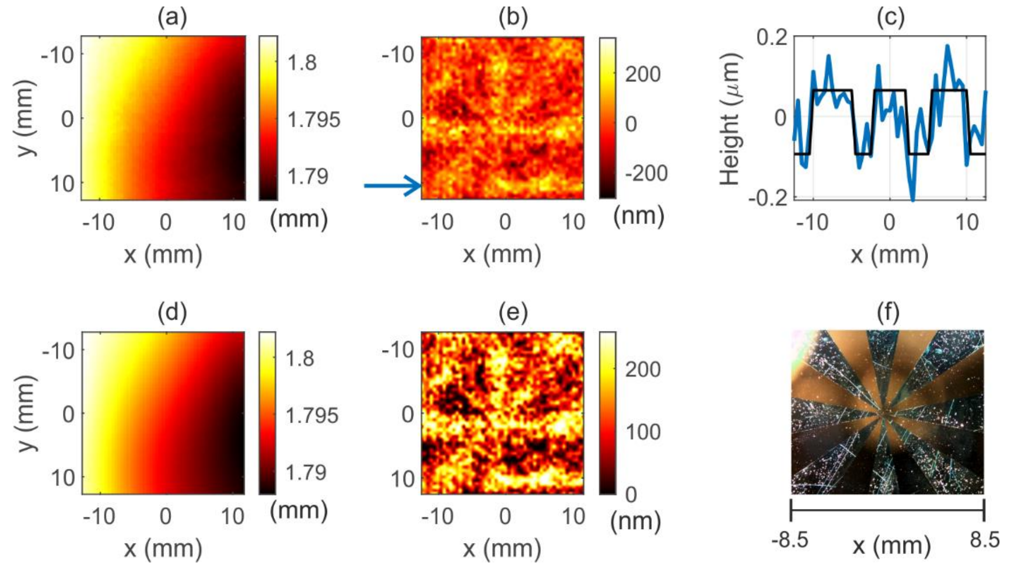

Fig. S10: a) Raw image data (51x51 pixels) of a 49 nm thick Siemens star of SiC on a 525 $\pm$ 5 µm thick silicon wafer (optical thickness of 1.787 mm) measured with an optical path length difference $\Delta(nd)$ = 2.4 cm. Warping of the substrate is clearly visible with an amplitude of about 5 µm. 49 nm thick Siemens star is not yet visible. b) Height profile after subtraction of warped background. c) Cross-section through the line indicated by the blue arrow in b). The black step profile with an optical height of 158.8 nm serves as a guide for the eye. The rms error of the optical thickness is 96.3 nm, corresponding to a physical thickness precision of about 31 nm. d) Warped background. e) Image enhanced by clipping and scaling height profile data in b). f) Optical micrograph Optical micrograph of the 49 nm SiC Siemens star.

References

[1] Liebermeister, L et al. Ultra-fast, high-bandwidth coherent CW THz spectrometer for non-destructive Testing. *Journal of Infrared, Millimeter, and Terahertz Waves* **40**, 288–296 (2019).

[2] Oden, J., et al. Imaging of broadband terahertz beams using an array of antenna-coupled microbolometers operating at room temperature. *Optics Express* **21**, 4817-4825 (2015).

[3] Boppel, S., et al. CMOS Integrated antenna-coupled field-effect transistors for the detection of radiation from 0.2 to 4.3 THz. *IEEE Transactions on Microwave Theory and Techniques* **60**, 3834-3843 (2012).

[4] Zolliker, P. and Hack, E. THz holography in reflection using a high resolution microbolometer array. *Optics Express* **23**, 10957-10967 (2015).

[5] Hodgson, N. & Weber H. *OPTICAL RESONATORS: FUNDAMENTALS, ADVANCED CONCEPTS and APPLICATIONS*. Springer-Verlag, London (1997).

[6] Naftaly, M., Molloy, J. F., Magnusson, B., Andreev, Y. M., & Lanskii, G. V. Silicon carbide—a high-transparency nonlinear material for THz applications. *Optics express* **24**, 2590-2595 (2016).

[7] Cataldo, G., et al. Infrared dielectric properties of low-stress silicon nitride. *Optics letters* **37**, 4200-4202 (2012).
